# Three-dimensional virtual reconstruction combined with the S.T.O.N.E. score improves outcomes of PCNL: a multicenter study

**DOI:** 10.3389/fsurg.2026.1880578

**Published:** 2026-09-28

**Authors:** Jia Yaodong, Tian Guolin, Li Yajie, Chen Yongfu, Feng Yuelong, Yang Hao, Li Jiaxin, Nie Lihong, Zhao Ruining

**Affiliations:** 1 Department of Urology, People’s Hospital of Ningxia Hui Autonomous Region, Yinchuan, Ningxia, China; 2 The Third Clinical Medical College, Ningxia Medical University, Yinchuan, Ningxia, China; 3 School of Basic Medical Sciences, Ningxia Medical University, Yinchuan, Ningxia, China

**Keywords:** 3D-VR, kidney stones, modified 3D-printed guide plate, PCNL, S.T.O.N.E. score, stone clearance rate

## Abstract

**Objective:**

To evaluate the impact of three-dimensional visualization virtual reconstruction (3D-VR) combined with the S.T.O.N.E. score on the efficacy of percutaneous nephrolithotomy (PCNL).

**Methods:**

A prospective cohort study enrolled patients with kidney stones from six centers between July 2022 and July 2025. Participants were divided into a conventional ultrasound-guided control group and a 3D-VR group based on the surgical strategy selected by patients after informed consent. The latter group was further subdivided into a Simple 3D-VR group and a 3D-VR-Template group. Each group was further categorized into low-to-moderate risk (S.T.O.N.E. score ≤ 9) and high-risk (S.T.O.N.E. score > 9) subgroups. Perioperative indicators were compared, and the relationship between scores and stone-free rates was analyzed using logistic regression. Stratified multivariable logistic regression analyses were performed to compare 3D-VR techniques against the conventional ultrasound-guided control group across risk-based subgroups (*P* < 0.05).

**Results:**

No baseline differences were observed among the three groups. Compared with the conventional ultrasound-guided control group, the Simple 3D-VR group alone demonstrated reduced intraoperative blood loss, shorter nephroscopic access establishment time, and shorter hospital stay (*P* < 0.05). The 3D-VR-Template group showed further reductions in these indicators and a significantly higher stone-free rate (*P* < 0.05). No statistically significant differences existed between the two 3D technology groups. S.T.O.N.E. score > 9 was an independent risk factor for low stone-free rate (OR = 2.31, 95%CI: 1.12–4.76, *P* = 0.02). Stratified analysis revealed that while the Simple 3D-VR technique doubled stone-free rate in low-to-moderate risk patients (OR = 2.07, 95%CI: 1.04–4.03, *P* = 0.039), the 3D-VR-Template technology provided a superior fourfold advantage (OR = 4.15, 95%CI: 1.07–6.14, *P* = 0.040) specifically in high-risk cases.

**Conclusion:**

Combining 3D-VR with S.T.O.N.E. scoring may optimize PCNL surgical planning, reduce intraoperative bleeding and shorten hospital stay, and may improve stone-free rate especially in patients with high-risk complex stones. Further well-designed controlled studies are warranted to confirm its clinical value.

## 1 Introduction

Kidney stones are a common urological disease, and the main clinical manifestations in patients are low back pain and hematuria. The main treatment is surgery. Percutaneous nephrolithotomy (PCNL) is a procedure proposed by Fernström et al. (1) in 1976, in which a small incision is made in the skin to create a channel to the renal calyx, renal pelvis, or upper part of the ureter, and then lithotripsy instruments are delivered into the kidneys through the channel to crush and remove the stone located in the upper part of the kidneys or ureters: lithotripsy and stone removal. Although PCNL shows many significant advantages over traditional open surgery (2), it is a complex procedure with many postoperative complications (3). Bleeding is the most common complication, which is directly related to estimated intraoperative blood loss and the selection of the puncture point (4). Therefore, accurately selecting the puncture point to localize the stone and minimize damage to the renal parenchyma has become a hot topic in PCNL research.Preoperative stone scoring systems are widely used to stratify surgical difficulty. The S.T.O.N.E. nephrolithometry score, a classic evaluation system proposed by Okhunov et al. (7), assesses stone complexity based on five two-dimensional CT parameters: stone size, tract length, hydronephrosis degree, number of involved calyces, and skin-to-stone distance. However, the score only relies on two-dimensional imaging information, ignores the three-dimensional spatial relationship between renal anatomy and calculi, and cannot provide personalized guidance for puncture path design, especially in complex high-risk cases.

3D technology has been rapidly advancing in recent years, and customized 3D-printed guides and models have been applied in clinical puncture assistance and surgical training for PCNL (6). Previous systematic reviews and meta-analyses (5) have demonstrated that 3D-assisted technology may improve puncture accuracy and reduce intraoperative bleeding, but most existing studies are single-center, small-sample investigations, and almost none conduct preoperative risk stratification for patients. It remains unclear which patients can obtain the greatest benefit from 3D technology, making it difficult to guide clinical individualized decision-making and cost control. In fact, the S.T.O.N.E. score and 3D-VR technology have natural complementary advantages: the S.T.O.N.E. score can preoperatively quantify stone complexity and screen high-risk patients with the greatest clinical demand, while 3D-VR can precisely compensate for the lack of spatial anatomical information in two-dimensional scoring and optimize personalized puncture path design. The combination of the two is expected to achieve the synergistic effect of “precise risk stratification + precise surgical operation”. However, to date, few studies have integrated S.T.O.N.E. risk stratification with 3D-VR technology, and the differential efficacy of different 3D-VR strategies in patients with different risk levels has not been clarified. Therefore, this prospective multicenter cohort study aimed to: (1) verify the predictive value of the S.T.O.N.E. score for perioperative outcomes of PCNL; (2) evaluate the efficacy of 3D-VR technology and modified 3D-printed guide template in improving perioperative outcomes of PCNL; (3) analyze the differential benefits of different 3D-VR strategies in patients with different S.T.O.N.E. risk stratification, so as to provide evidence for individualized surgical decision-making.

## 2 Materials and methods

### 2.1 General information

This study adopted a prospective cohort study and selected some patients who were diagnosed with kidney stones and underwent percutaneous nephrolithotomy at the General Hospital of Ningxia Medical University, the People's Hospital of Ningxia Hui Autonomous Region, the People's Hospital of Wuzhong City, the People's Hospital of Shizuishan City, the People's Hospital of Yanchi County, and the People's Hospital of Huinong District from July 2022 to July 2025. All patients were consecutively enrolled and divided into the conventional ultrasound-guided conventional ultrasound-guided control group and the 3D-VR-assisted group according to the surgical plan selected by patients after informed consent. The 3D-VR group was further subdivided into the Simple 3D-VR subgroup and the 3D-VR-Template subgroup. Considering the high surgical difficulty and stronger clinical demand for high-risk patients, the 3D-printed guide template was mainly applied to patients with S.T.O.N.E. score > 9 under the premise of voluntary choice of patients. Patients were stratified by S.T.O.N.E. score criteria, each group of patients was divided into low-to-moderate risk (S.T.O.N.E. score ≤ 9) and high risk (S.T.O.N.E. score > 9). The details of the S.T.O.N.E scoring system are listed in Table 1, the hydronephrosis grading criteria are provided in Table 2, and the S.T.O.N.E. nephrolithometry scoring system based on preoperative imaging is shown in Figure 1.

**Table 1:** S.T.O.N.E scoring system.

Item	1 point	2 point	3 point	4 point
S	0–399	400–799	800–1,599	≥1,600
T	≤100	>100	—	—
O	No or mild hydronephrosis	Moderate or severe hydronephrosis	—	—
N	≤1	2–3	Staghorn calculus	—
E	≤950	>950	-	—

**Table 2:** Hydronephrosis grading criteria.

Grade	Criteria
Grade 0	No pelvicalyceal dilation
Grade 1 (mild)	Mild pelvic dilation; calyces not dilated
Grade 2 (mild)	Slight dilation of both pelvis and calyces; normal morphology preserved
Grade 3 (moderate)	Moderate dilation of pelvis and calyces with morphologic distortion and cortical thinning
Grade 4 (severe)	“Ballooned” kidney; pelvicalyceal boundaries indistinct; renal parenchymal atrophy
	

**Figure 1:**
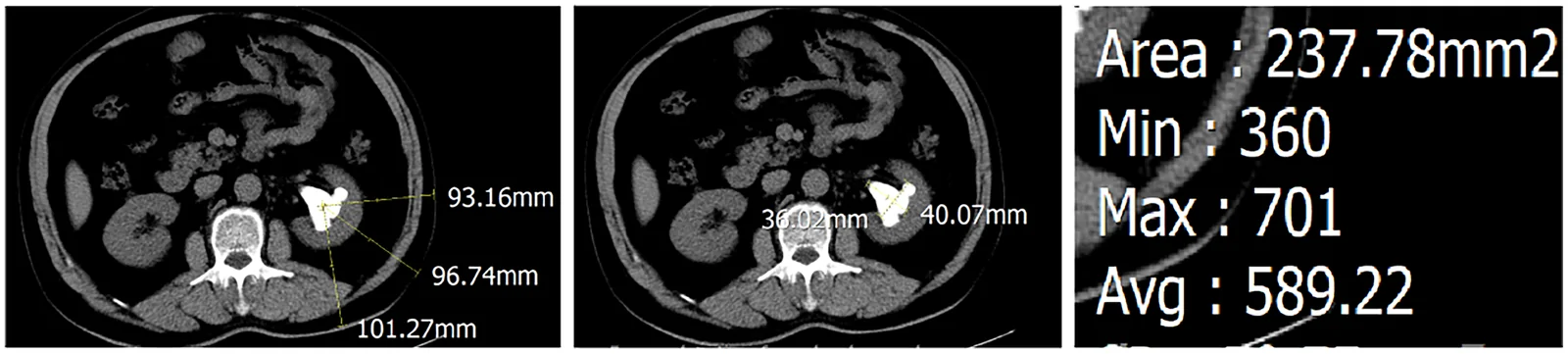
S.T.O.N.E. nephrolithometry scoring system based on preoperative imaging.

### 2.2 3D reconstruction and guide plate fabrication

The patient was placed in a prone position, with the waist and buttocks elevated to the intraoperative level. Electrode patches were pasted at the intersection of the 12th rib and the lower scapular line on the affected side and beside the L1-L2 vertebrae as intraoperative positioning markers, and a CTU scan of the urinary system was performed. The obtained DICOM images were imported into the E-3D Digital Medical Modeling and Design System for personalized three-dimensional reconstruction (13). The reconstructed model can be rotated at any angle to clearly display the spatial anatomical relationship of the kidney and calculi (14), which facilitates preoperative puncture path planning. In the E3D software, the contour of the fitting guide plate is drawn based on the skin shape in the surgical area, and the positioning points are set in conjunction with the electrode sheet's position. The main curved surface is retained, and the curved surface of the puncture area is appropriately reduced to form a “D” -shaped hollowed-out design guide plate model. This design not only preserves the fixed puncture channel on the template, but also reserves operating space for the intraoperative ultrasound probe, realizing dual positioning assistance of 3D template and ultrasound, which helps to improve puncture accuracy. The puncture point was designed as a cylinder with a diameter of 1.2 mm and a height of 1 mm. According to the SVOF principle (short distance, vertex, obtuse angle, and fornix principle) (16), the posterior group of the middle calyces was selected as the target calyx for puncture path planning. This area is close to the Brodel's avascular plane of the kidney, which can reduce the risk of intraoperative parenchymal injury and bleeding, and provides a larger working space for the nephroscope, The puncture path was designed to be approximately parallel to the long axis of the target renal calyx. The skin puncture point was located 1 to 1.5 cm below the lower edge of the 12th rib, ensuring that the path avoided adjacent organs such as the spleen and liver. Final model data were imported into a 3D stereolithography printer (Suzhou Zhongrui Zhichuang 3D Technology Co., LTD.), which uses photosensitive resin materials with good thermal stability, low linear shrinkage rate, and excellent mechanical properties for printing. This material can maintain dimensional stability after sterilization, with good biosafety, and the printing cycle and cost are more controllable than traditional materials, which is conducive to clinical promotion, Models were printed layer-by-layer via photocuring, as shown in Figure 2. Some of the 3D-printed navigation templates, systematically manufactured and applied to 3D reconstruction of patients in multiple directions, are shown in Figure 3. The remaining schematic diagrams and demonstrations are available in Supplementary Material 1.

**Figure 2:**
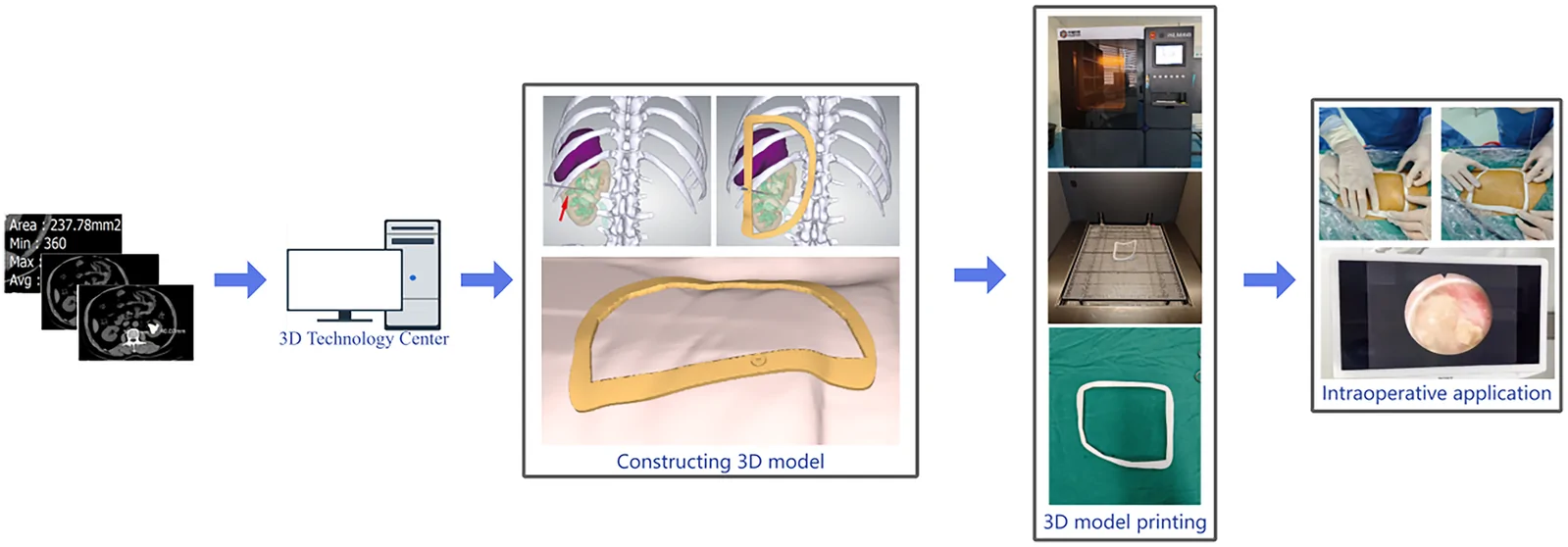
Guide plate fabrication and intraoperative application.

**Figure 3:**
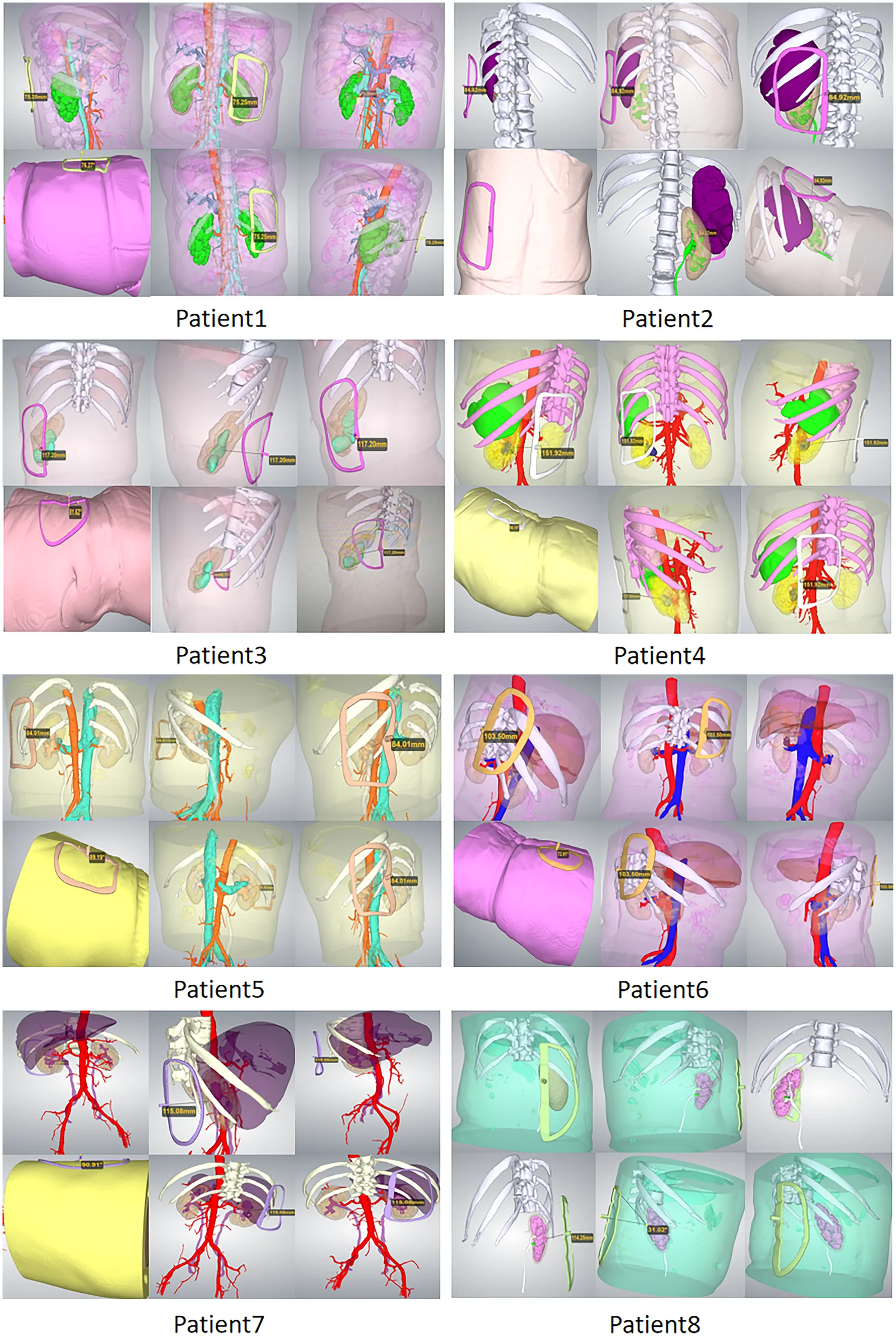
Shows a multi-directional schematic diagram of 3D-printed navigation templates and 3D reconstruction for some patients.

### 2.3 Surgical methods

After anesthesia induction, patients were placed in the lithotomy position. First, a 5F ureteral catheter is inserted into the affected ureter, and 0.9% normal saline is continuously infused. The conventional ultrasound-guided control group located the target with an ultrasound probe and performed a percutaneous nephrostomy puncture using a sterile 18-G needle. In the 3D-VR-Template group, the sterilized guide template was placed the disinfected guide plates in the preset area and, with ultrasound assistance, punctures along the preset path of the guide plates. After successful puncture, a J-type safety guide wire was inserted, and the fascia dilator was used to expand to F21. Then, the F24 lens sheath was pushed in, and an F20.8 nephroscope was placed for observation. The time from initial ultrasound scanning of the renal region to successful placement of the nephroscope sheath was recorded as the nephroscope access establishment time. Subsequently, a routine PCNL was performed, and the total operative time was recorded. The intraoperative blood loss was determined by the cyanmethemoglobin method. One week postoperatively, kidney-ureter-bladder (KUB) radiography was uniformly performed in all patients to assess stone clearance status, instead of selective use. Stone-free status was defined as no visible residual fragments or residual fragments ≤4 mm in maximum diameter on KUB; this threshold is widely adopted in PCNL clinical studies and guidelines, as fragments of this size are generally considered spontaneously passable and do not require secondary surgical intervention. Fragments >4 mm were defined as residual stone.

All postoperative KUB images were independently reviewed by two senior urologists who were blinded to patient group allocation and surgical procedures. Discrepancies in stone residual judgment were resolved by adjudication with a third senior urologist. Standardized abdominal radiography protocols were applied for all patients to minimize imaging bias.

### 2.4 Statistical processing

Measurement data were expressed as mean ± standard deviation, and comparisons between two groups were performed using independent-samples *t*-test. Qualitative data were presented as counts and percentages, and comparisons between groups were performed using chi-square tests. Mann–Whitney *U*-test was used for data that did not conform to normal distribution. A two-sided *P* value ≤ 0.05 was considered statistically significant.

No *a priori* sample size calculation was performed, as this was a prospective observational cohort study with consecutive patient enrollment. A *post-hoc* power analysis for the primary outcome (stone-free rate) was conducted using PASS 15.0 software. For the comparison between the 3D-VR-Template group and the conventional ultrasound-guided control group in high-risk patients, the statistical power reached 82% at a two-sided *α* level of 0.05, which was considered acceptable for the primary endpoint analysis.

Given the observational design with non-randomized group allocation, potential selection bias and confounding factors may affect the outcome evaluation. To independently estimate the net effect of 3D-VR techniques on stone-free rate, multivariable logistic regression models were established with adjustment for baseline confounders, including maximum stone cross-sectional area, degree of hydronephrosis, number of involved calyces, and patient age.

First, univariable and multivariable logistic regression models were used to analyze the association between the S.T.O.N.E. score (and its subitems) and stone residual rate. Independent variables included intervention types (categorical variables: conventional ultrasound-guided control group, Simple 3D-VR group, 3D-VR-Template group), S.T.O.N.E. risk stratification (binary variables: low-to-moderate risk ≤9 points vs. high risk >9 points), and the above-mentioned baseline covariates.

Furthermore, stratified multivariable logistic regression analyses were performed to compare the efficacy of different 3D-VR strategies against the conventional ultrasound-guided control group across S.T.O.N.E. risk subgroups. The stratified analyses by S.T.O.N.E. risk level were pre-specified in accordance with the study hypothesis, aiming to explore the differential efficacy of 3D-VR strategies across different risk subgroups, rather than *post-hoc* multiple testing. No adjustment for multiple comparisons was performed in this study, and the subgroup analysis results were interpreted as exploratory findings.

## 3 Results

### 3.1 Case collection

A total of 156 consecutive patients with renal calculi who met the inclusion criteria were enrolled in this prospective cohort study. According to the surgical strategy selected by patients after full informed consent, all participants were allocated to the conventional ultrasound-guided conventional ultrasound-guided control group (*n* = 78) or the 3D-VR-assisted group (*n* = 78). The 3D-VR group was further subdivided into the Simple 3D-VR subgroup (*n* = 59, 3D virtual reconstruction only) and the 3D-VR-Template subgroup (*n* = 19, 3D reconstruction combined with 3D-printed puncture guide template).

All patients were stratified by the S.T.O.N.E. scoring system into low-to-moderate risk (score ≤ 9) and high-risk (score > 9) subgroups. In the conventional ultrasound-guided control group, 30 cases were classified as low-to-moderate risk and 48 as high-risk. In the Simple 3D-VR group, 31 cases were low-to-moderate risk and 28 were high-risk. All 19 patients in the 3D-VR-Template group were high-risk cases, as the 3D-printed guide template was mainly recommended for patients with complex high-risk stones under voluntary choice.

### 3.2 General information

Baseline comparability was verified by stratified analysis according to S.T.O.N.E. risk grade. There were no statistically significant differences in baseline demographic and clinical indicators between groups within the same risk stratum (all *P* > 0.05), as detailed in Tables 3–6.

**Table 3:** Comparison of baseline characteristics between the conventional ultrasound-guided control group and simple 3D-VR group in low-to-moderate risk patients (S.T.O.N.E. score ≤ 9).

Item	Conventional ultrasound-guided control group (*n* = 30)	Simple 3D-VR group (*n* = 31)	*t*/*χ*^2^	*P*
Age	52.90 ± 11.56	51.84 ± 9.92	0.385	0.701
BMI	23.82 ± 2.34	25.23 ± 3.23	−1.960	0.055
Stone location			0.031	0.860
Right	10 (33.33)	11 (35.48)		
Left	20 (66.67)	20 (64.52)		
Symptomatic vs. asymptomatic			0.024	0.878
Asymptomatic	12 (40.00)	13 (41.94)		
Symptomatic	18 (60.00)	18 (58.06)		
Sex			0.075	0.785
Male	25 (83.88)	25 (80.65)		
Female	5 (16.67)	6 (19.35)		

**Table 4:** Comparison of baseline characteristics between the conventional ultrasound-guided control group and simple 3D-VR group in high-risk patients (S.T.O.N.E. score > 9).

Item	Conventional ultrasound-guided control group (*n* = 48)	Simple 3D-VR group (*n* = 28)	*t*/*χ*^2^	*P*
Age	50.73 ± 11.86	55.18 ± 8.77	−1.73	0.088
BMI	25.44 ± 3.04	25.00 ± 2.73	0.62	0.534
Stone location			1.29	0.257
Right	16 (33.33)	13 (46.43)		
Left	32 (66.67)	15 (53.57)		
Symptomatic vs. asymptomatic			1.00	0.318
Asymptomatic	21 (43.75)	9 (32.14)		
Symptomatic	27 (56.25)	19 (67.86)		
Sex			0.98	0.322
Male	39 (81.25)	20 (71.43)		
Female	9 (18.75)	8 (28.57)		

**Table 5:** Comparison of baseline characteristics between the conventional ultrasound-guided control group and 3D-VR-template group in high-risk patients (S.T.O.N.E. score > 9).

Item	Conventional ultrasound-guided control group (*n* = 48)	3D-VR-template group (*n* = 19)	*t*/*χ*^2^	*P*
Age	50.73 ± 11.86	54.89 ± 10.71	−1.33	0.188
BMI	25.44 ± 3.04	26.38 ± 2.83	−1.16	0.249
Stone location			0.07	0.785
Right	16 (33.33)	7 (36.84)		
Left	32 (66.67)	12 (63.16)		
Symptomatic vs. asymptomatic			0.07	0.788
Asymptomatic	21 (43.75)	9 (47.37)		
Symptomatic	27 (56.25)	10 (52.63)		
Sex			1.56	0.212
Male	39 (81.25)	12 (63.16)		
Female	9 (18.75)	7 (36.84)		

**Table 6:** Comparison of baseline characteristics between the simple 3D-VR group and 3D-VR-template group in high-risk patients (S.T.O.N.E. score > 9).

Item	Simple 3D-VR group (*n* = 28)	3D-VR-template group (*n* = 19)	*t*/*χ*^2^	*P*
Age	55.18 ± 8.77	54.89 ± 10.71	0.10	0.921
BMI	25.00 ± 2.73	26.38 ± 2.83	−1.67	0.102
Stone location			0.43	0.514
Right	13 (46.43)	7 (36.84)		
Left	15 (53.57)	12 (63.16)		
Symptomatic vs. asymptomatic			1.11	0.292
Asymptomatic	9 (32.14)	9 (47.37)		
Symptomatic	19 (67.86)	10 (52.63)		
Sex			0.36	0.551
Male	20 (71.43)	12 (63.16)		
Female	8 (28.57)	7 (36.84)		

### 3.3 Comparison of perioperative clinical data between low-to-moderate risk and high-risk patients with S.T.O.N.E. scores in the conventional ultrasound-guided control group

Comparison of perioperative indicators between the two groups showed that in the conventional ultrasound-guided control group, the maximum cross-sectional area of stones in the low-to-moderate riskgroup was smaller than that in the high-risk group (7.34 ± 6.26 cm^2^ vs. 12.74 ± 9.65 cm^2^), the operation time was shorter than that in the high-risk group (1.33 ± 0.33 h vs. 1.68 ± 0.65 h). The stone-free rate was better than that in the high-risk group (90.00% vs. 56.25%), and the middle calyx was the most common puncture target in both groups. The differences were statistically significant (*P* < 0.05), No statistically significant differences were observed between groups in intraoperative estimated blood loss, time to establish nephroscopic access, length of hospital stay, magnitude of changes in renal function, magnitude of changes in hemoglobin, infection rate, and number of days with nephrostomy tube retention (*P* > 0.05), as shown in Table 7.

**Table 7:** Comparison of perioperative data between low-to-moderate risk and high-risk patients in the S.T.O.N.E. score of the conventional ultrasound-guided control group.

Item	Low-to-moderate risk group (*n* = 30)	High-risk group (*n* = 48)	*t*/*χ*^2^	*P*
Maximum cross-sectional area of calculi	7.34 ± 6.26	12.74 ± 9.65	2.72^a^	0.008**
Operation time	1.33 ± 0.33	1.68 ± 0.65	3.15^a^	0.002**
Estimated estimated intraoperative blood loss	59.60 ± 30.13	55.11 ± 37.10	−0.56^a^	0.597
Nephroscope access establishment time	22.50 ± 9.48	22.67 ± 10.96	0.07^a^	0.945
Hospital stay	10.87 ± 2.75	11.08 ± 2.45	0.36^a^	0.718
Change range of renal function	0.30(−5.15,5.90)	2.90(−2.60,8.43)	−0.90^c^	0.366
Change range of hemoglobin	−8.00(−14.50,0.00)	−8.00(−15.00,0.50)	−0.06^c^	0.955
Stone-free rate			9.87^b^	0.002**
Clearance	27 (90.00)	27 (56.25)		
Residue	3 (10.00)	21 (43.75)		
Puncture calyx			—	0.019*
Upper calyx	3 (10.00)	0 (0.00)		
Middle calyx	26 (86.67)	48 (100.00)		
Lower calyx	1 (3.33)	0 (0.00)		
Infection rate			2.09^b^	0.149
No infection	20 (66.67)	24 (50.00)		
With infection	10 (33.33)	24 (50.00)		
Retention days of nephrostomy tube			—	0.341
2 days	3 (3.85)	1 (2.08)		
3 days	27 (90.00)	42 (87.50)		
4 days	1 (3.33)	5 (10.42)		

“*” indicates *P* < 0.05 when comparing the low-to-moderate risk group with the high-risk group; “**” indicates *P* < 0.01 when comparing the low-to-moderate risk group with the high-risk group. ^a^*t*-test, ^b^chi-square tests, ^c^Mann–Whitney test, —: Fisher exact.

### 3.4 Comparison of perioperative clinical data between the conventional ultrasound-guided control group and the simple 3D-VR group

Comparison of perioperative indicators between groups showed that the estimated estimated intraoperative blood loss in the simple 3D-VR group was less than that in the conventional ultrasound-guided control group (43.08 ± 30.71 mL vs. 56.84 ± 34.45 mL). The establishment time of the nephroscopic access was shorter than that in the conventional ultrasound-guided control group (16.41 ± 7.20 min vs. 22.60 ± 10.35 min). The length of hospital stay was shorter than that of the conventional ultrasound-guided control group (9.14 ± 2.69 d vs. 11.00 ± 2.55 d). The differences were statistically significant (*P* < 0.05), No statistically significant differences were observed between groups in the maximum cross-sectional area of stones, operation time, magnitude of changes in renal function, magnitude of changes in hemoglobin, stone-free rate, infection rate, and duration of nephrostomy tube retention (*P* > 0.05), as shown in Table 8.

**Table 8:** Comparison of perioperative data between the conventional ultrasound-guided control group and the simple 3D-VR group.

Item	Conventional ultrasound-guided control group (*n* = 78)	Simple 3D-VR group (*n* = 59)	*t*/*Z*/*χ*^2^	*P*
Maximum cross-sectional area of calculi	10.66 ± 8.87	8.94 ± 8.37	1.16^a^	0.250
Operation time	1.55 ± 0.58	1.46 ± 0.56	0.88^a^	0.382
Estimated estimated intraoperative blood loss	56.84 ± 34.45	43.08 ± 30.71	2.42^a^	0.017*
Nephroscope access establishment time	22.60 ± 10.35	16.41 ± 7.20	4.13^a^	0.01**
Hospital stay	11.00 ± 2.55	9.14 ± 2.69	4.14^a^	0.01**
Change range of renal function	2.55 ± 9.67	1.67 ± 9.32	0.54^a^	0.592
Change range of hemoglobin	−8.00 (−15.00,0.00)	−9.00 (−11.00,−1.00)	−0.46^c^	0.645
Stone-free rate			1.89^b^	0.170
Clearance	54 (69.23)	47 (79.66)		
Residue	24 (30.77)	12 (20.34)		
Puncture calyx			—	0.097
Upper calyx	3 (3.85)	7 (11.86)		
Middle calyx	74 (94.87)	49 (83.05)		
Lower calyx	1 (1.28)	3 (5.08)		
Infection rate			0.29^b^	0.588
No infection	44 (56.41)	36 (61.02)		
With infection	34 (43.59)	23 (38.98)		
Retention days of nephrostomy tube			—	0.074
2 days	3 (3.85)	0 (0.00)		
3 days	69 (88.46)	58 (98.31)		
4 days	6 (7.69)	1 (1.69)		

“*” indicates *P* < 0.05 when comparing the low-to-moderate risk group with the high-risk group; “**” indicates *P* < 0.01 when comparing the low-to-moderate risk group with the high-risk group, ^a^*t*-test, ^b^chi-square tests, ^c^Mann–Whitney test. —: Fisher exact.

### 3.5 Comparison of perioperative clinical data between the conventional ultrasound-guided control group and the simple 3D-VR group, as well as the low-to-moderate risk and high-risk patient groups in the S.T.O.N.E. score

Stratified analysis by risk grade showed the following results: 1. The establishment time of nephroscopic access in the simple 3D-VR low-risk patient group was shorter than that in the Low-to-moderate risk subgroup of conventional ultrasound-guided control group (16.90 ± 7.20 min vs. 22.50 ± 9.48 min). The hospital stay was shorter than that in the Low-to-moderate risk subgroup of conventional ultrasound-guided control group (8.74 ± 1.93d vs. 10.87 ± 2.75d). The Estimated estimated intraoperative blood loss was smaller than that of the Low-to-moderate risk subgroup of conventional ultrasound-guided control group (41.35 ± 31.31 g/L vs. 59.60 ± 30.13 g/L). The differences were all statistically significant (*P* < 0.05). No statistically significant differences were observed between groups in the maximum cross-sectional area of stones, operation time, intraoperative estimated blood loss, magnitude of changes in renal function, stone-free rate, infection rate, and duration of nephrostomy tube retention (*P* > 0.05). Among patients at low-to-moderate risk, the use of three-dimensional virtual reconstruction technology facilitates the establishment of nephroscopic access. These differences were accompanied by significantly shorter hospital stay, with no significant between-group differences in hemoglobin change. 2. The establishment time of nephroscopic access in the simple 3D-VR high-risk patient group was shorter than that in the High-risk subgroup of conventional ultrasound-guided control group (15.86 ± 7.29 min vs. 22.67 ± 10.96 min), and the hospital stay was shorter than that in the High-risk subgroup of conventional ultrasound-guided control group (9.57 ± 3.32 d vs. 11.08 ± 2.45d). The differences were all statistically significant (*P* < 0.05). No statistically significant differences were observed between groups in the maximum cross-sectional area of stones, operation time, intraoperative estimated blood loss, magnitude of changes in renal function, magnitude of changes in hemoglobin, stone-free rate, infection rate, and duration of nephrostomy tube retention (*P* > 0.05). In high-risk patients, the application of three-dimensional visualization and virtual reconstruction technology has advantages in terms of time required for nephroscopic access and hospital stay, as shown in Table 9. However, given the limited sample size of the 3D-VR-Template subgroup, the above efficacy findings should be interpreted as exploratory, and further large-scale multicenter studies are required to confirm these results.

**Table 9:** Shows the comparison of perioperative data between the conventional ultrasound-guided control group and the simple 3D-VR group, as well as between the low-risk and high-risk patient groups, in terms of S.T.O.N.E. scores.

Item	Low-to-moderate risk subgroup of conventional ultrasound-guided control group (*n* = 30)	Low-to-moderate risk subgroup of simple 3D-VR group (*n* = 31)	*t*/*χ*^2^	*P*	High-risk subgroup of conventional ultrasound-guided control group (*n* = 48)	High-risk subgroup of simple 3D-VR group (*n* = 28)	*t*/*χ*^2^	*P*
Maximum cross-sectional area of calculi	7.34 ± 6.26	5.83 ± 5.99	0.96^a^	0.339	12.74 ± 9.65	12.38 ± 9.33	0.16^a^	0.875
Operation time	1.33 ± 0.33	1.40 ± 0.55	−0.60^a^	0.550	1.68 ± 0.65	1.53 ± 0.57	1.04^a^	0.300
Estimated estimated intraoperative blood loss	59.60 ± 30.13	41.35 ± 31.31	2.32^a^	0.024*	55.11 ± 37.10	45.00 ± 30.49	1.22^a^	0.226
Nephroscope access establishment time	22.50 ± 9.48	16.90 ± 7.20	2.60^a^	0.012*	22.67 ± 10.96	15.86 ± 7.29	2.93^a^	0.005**
Hospital stay	10.87 ± 2.75	8.74 ± 1.93	3.50^a^	<.001**	11.08 ± 2.45	9.57 ± 3.32	2.27^a^	0.026*
Change range of renal function	0.84 ± 8.34	3.21 ± 8.68	−1.09^a^	0.282	3.62 ± 10.36	−0.03 ± 9.85	1.51^a^	0.136
Change range of hemoglobin	−8.11 ± 9.65	−5.97 ± 8.81	−0.91^a^	0.369	−7.71 ± 12.14	−8.29 ± 8.57	0.22^a^	0.826
Stone-free rate			0.00^b^	1			6.28^b^	0.189
Clearance	27 (90.00)	27 (87.10)			27 (56.25)	20 (71.43)		
Residue	3 (10.00)	4 (12.90)			21 (43.75)	8 (28.57)		
Puncture calyx			—	0.700			2.90^b^	0.089
Upper calyx	3 (10.00)	4 (12.90)			0 (0.00)	3 (10.71)		
Middle calyx	26 (86.67)	24 (77.42)			48 (100.00)	25 (89.29)		
Lower calyx	1 (3.33)	3 (9.68)						
Infection rate			0.19	0.662			0.82^b^	0.366
No infection	20 (66.67)	19 (61.29)			24 (50.00)	17 (60.71)		
With infection	10 (33.33)	12 (38.71)			24 (50.00)	11 (39.29)	—	0.622
Retention days of nephrostomy tube			—	0.113	1 (2.08)	0 (0.00)		
2 days	2 (6.67)	0 (0.00)			42 (87.50)	27 (96.43)		
3 days	27 (90.00)	31 (100.00)			5 (10.42)	1 (3.57)		
4 days	1 (3.33)	0 (0.00)						

“*” indicates that *P* < 0.05 compared with the simple 3D-VR group in the conventional ultrasound-guided control group, and “**” indicates that *P* < 0.01 compared with the simple 3D-VR group in the conventional ultrasound-guided control group. ^a^*t*-test, ^b^chi-square tests, —: Fisher exact.

### 3.6 Comparison of perioperative clinical data between the high-risk subgroup of conventional ultrasound-guided control group and the high-risk patient group with 3D-VR-template group in patients with S.T.O.N.E. score

Comparison of perioperative indicators between groups showed that the 3D-VR-Template group had less estimated estimated intraoperative blood loss than the high-risk subgroup of conventional ultrasound-guided control group (40.90 ± 23.66 mL vs. 63.23 ± 29.84 mL). The establishment time for nephroscopic access was shorter than that of the high-risk subgroup of conventional ultrasound-guided control group (14.37 ± 5.08 min vs. 22.67 ± 10.96 min). The duration of hospitalization was shorter than that of the high-risk subgroup of conventional ultrasound-guided control group (8.42 ± 1.22 d vs. 11.08 ± 2.45 d). The stone-free rate was higher than that of the high-risk subgroup of conventional ultrasound-guided control group (84.21% vs. 56.25%), and the difference was statistically significant (*P* < 0.05). No statistically significant differences were observed between groups in the maximum cross-sectional area of stones, operation time, magnitude of changes in renal function, magnitude of changes in hemoglobin, infection rate, and duration of nephrostomy tube retention (*P* > 0.05). Among high-risk patients, the three-dimensional visualization and virtual reconstruction technology, and the construction of 3D-printed guide plates, offer clear advantages and greater benefits, as shown in Table 10.

**Table 10:** Comparison of perioperative data of high-risk patients with S.T.O.N.E. scores between the high-risk subgroup of conventional ultrasound-guided control group and the 3D-VR-Template group.

Item	High-risk subgroup of conventional ultrasound-guided control group (*n* = 48)	3D-VR-template group (*n* = 19)	*t*/*χ*^2^	*P*
Maximum cross-sectional area of calculi	12.74 ± 9.65	11.35 ± 7.65	0.56^a^	0.576
Operation time	1.68 ± 0.65	1.40 ± 0.43	1.76^a^	0.083
Estimated estimated intraoperative blood loss	63.23 ± 29.84	40.90 ± 23.66	2.92^a^	0.005**
Nephroscope access establishment time	22.67 ± 10.96	14.37 ± 5.08	4.22^a^	<.001**
Hospital stay	11.08 ± 2.45	8.42 ± 1.22	5.91^a^	<.001**
Change range of renal function	3.62 ± 10.36	1.95 ± 13.33	0.55^a^	0.586
Change range of hemoglobin	−7.98 ± 12.00	−6.26 ± 6.35	−0.59^a^	0.557
Stone-free rate			9.91^b^	0.031*
Clearance	27 (56.25)	16 (84.21)		
Residue	21 (43.75)	3 (15.79)		
Puncture calyx			—	0.490
Middle calyx	47 (97.92)	18 (94.74)		
Lower calyx	1 (2.08)	1 (5.26)		
Infection rate			0.04^b^	0.846
No infection	24 (50.00)	10 (52.63)		
With infection	24 (50.00)	9 (47.37)		
Retention days of nephrostomy tube			—	0.091
2 days	1 (2.08)	2 (10.53)		
3 days	42 (87.50)	17 (89.47)		
4 days	5 (10.42)	0 (0.00)		

“*” indicates that *P* < 0.05 when comparing the high-risk subgroup of conventional ultrasound-guided control group with the 3D-VR-guided group, and “**” indicates that *P* < 0.01 when comparing the high-risk subgroup of conventional ultrasound-guided control group with the 3D-VR-guided group. ^a^*t*-test, ^b^chi-square tests, —: Fisher exact.

### 3.7 Comparison of perioperative clinical data of high-risk patients with S.T.O.N.E. scores between the simple 3D-VR group and the 3D-VR-Template group

Comparison of perioperative indicators between the two high-risk groups showed that there were no statistically significant differences between the two groups in terms of estimated intraoperative blood loss, the time for establishing the nephroscopic access, the length of hospital stay, the maximum cross-sectional area of the stone, the operation time, the range of changes in renal function, the range of changes in hemoglobin, the infection rate, and the retention days of the nephrostomy tube (*P* > 0.05). The stone-free rate of the 3D-VR guide plate group was slightly higher than that of the simple 3D-VR group, but the difference did not reach statistical significance (*P* > 0.05). See Table 11.

**Table 11:** Comparison of perioperative data of high-risk patients with S.T.O.N.E. scores between the simple 3D-VR group and the 3D-VR-template group.

Item	Simple 3D virtual reconstruction group (*n* = 28)	3D-VR-template group (*n* = 19)	*t*/*χ*^2^	*P*
Maximum cross-sectional area of calculi	12.38 ± 9.33	11.35 ± 7.65	0.40^a^	0.691
Operation time	1.53 ± 0.57	1.40 ± 0.43	0.85^a^	0.399
Estimated estimated intraoperative blood loss	45.00 ± 30.49	40.90 ± 23.66	0.49^a^	0.624
Nephroscope access establishment time	15.86 ± 7.29	14.37 ± 5.08	0.77^a^	0.445
Hospital stay	9.57 ± 3.32	8.42 ± 1.22	1.44^a^	0.156
Change range of renal function	−0.03 ± 9.85	1.95 ± 13.33	−0.59^a^	0.561
Change range of hemoglobin	−8.29 ± 8.57	−6.26 ± 6.35	−0.88^a^	0.385
Stone-free rate			0.44^b^	0.506
Clearance	20 (71.43)	16 (84.21)		
Residue	8 (28.57)	3 (15.79)		
Puncture calyx			-	0.147
Upper calyx	3 (10.71)	0 (0.00)		
Middle calyx	25 (89.29)	18 (94.74)		
Lower calyx	0 (0.00)	1 (5.26)		
Infection rate			0.30^b^	0.582
No infection	17 (60.71)	10 (52.63)		
With infection	11 (39.29)	9 (47.37)		
Retention days of nephrostomy tube			—	0.158
2 days	27 (96.43)	17 (89.47)		
3 days	1 (3.57)	0 (0.00)		
4 days	0 (0.00)	2 (10.53)		

a *t*-test, ^b^chi-square tests, —: Fisher exact.

### 3.8 Correlation analysis between S.T.O.N.E score and stone clearance rate

Logistic regression analysis showed that the total S.T.O.N.E. score, the cross-sectional area of the stone (S), the length of the pathway (T), and the number of involved renal calyces (*N*) were correlated with the residual rate of the stone (*P* *<* 0.05), as shown in Table 12.

**Table 12:** Relationship between the total S.T.O.N.E. score and each detailed score and the residual stone rate.

Variable	*P*	OR (95%CI)
S.T.O.N.E	<0.001	1.54 (1.22–1.95)
S		
1		Reference
2	0.008	5.53 (1.55–19.79)
3	0.011	6.06 (1.51–24.36)
4	0.002	26.67 (3.39–209.94)
T		
1		Reference
2	0.003	3.51 (1.53–8.03)
O		
1		Reference
2	0.691	0.85 (0.38–1.88)
N		
1		Reference
2	0.567	1.30 (0.52–3.26)
3	0.023*	2.94 (1.16–7.45)
E		
1		Reference
2	0.188	1.68 (0.78–3.64)

“*” indicates that the correlation analysis between the variable and the residual stone rate is *P* < 0.05.

### 3.9 Analysis of the effect of 3D technology in high and low risk subgroups

To accurately evaluate the value of 3D-VR technology in patients with stones of different complexities, we conducted a hierarchical logistic regression analysis (Table 13). Among low-to-moderate risk patients, the Simple 3D-VR technique independently increased the stone-free rate by approximately 2 times (OR = 2.07, 95%CI: 1.04–4.03, *P* = 0.039). However, in high-risk patients, Simple 3D-VR showed an improving trend but did not reach statistical significance (OR = 1.94, 95%CI: 0.72–5.28, *P* = 0.192); the 3D-VR-Template technology demonstrated a significant advantage, increasing the stone-free rate to more than four times that of the conventional ultrasound-guided control group (OR = 4.15, 95%CI: 1.07–6.14, *P* = 0.040). Notably, this significant advantage of the 3D-VR-Template in high-risk patients was derived from a small sample subgroup, and thus should be regarded as an exploratory finding. This result clearly indicates that 3D-printed solid guide plates play a crucial role in enhancing the stone-free effect of complex and high-risk PCNL stones.

**Table 13:** Logistic regression analysis results of stone-free rate by 3D-VR technology in different S.T.O.N.E. risk stratification subgroups.

Risk stratification	Variable	OR (95%CI)	*P*
Low to medium-risk (≤9 points)	Simple 3D-VR	2.07 (1.04–4.03)	0.039*
High-risk (9 points)	Simple 3D-VR	1.94 (0.72–5.28)	0.192
	3D-VR-Template	4.15 (1.07–6.14)	0.04*

“*” indicates that the correlation analysis between the variable and the residual stone rate is *P* < 0.05.

## 4 Discussion

Renal calculi, also known as urinary calculi or urinary tract calculi, are common diseases of the urinary system. Epidemiological data show that approximately 10% of adults in the United States suffer from renal calculi, and the incidence of renal calculi is on the rise worldwide (8). For patients with renal calculi, symptoms depend mainly on the size and location of the calculi, as well as whether there is obstruction (9). When calculi are small, most patients have no obvious symptoms, and many are diagnosed during physical examination. When calculi are large and obstructive, most patients experience symptoms such as renal colic, hematuria, hydronephrosis, and urinary tract infection. Without timely intervention, the condition may progress to severe complications such as obstructive renal failure and urosepsis, which are life-threatening. Therefore, for large renal calculi (≥2 cm), scholars agree that surgery is the preferred treatment method (10). Since percutaneous nephrolithotripsy (PCNL) was introduced in 1976 (1), after years of unremitting attempts and explorations by scholars worldwide, it has now become the preferred surgical method for large and complex upper urinary tract calculi. However, the complications of PCNL remain a major clinical problem. For example, if massive estimated intraoperative blood loss is not effectively controlled, severe cases may require immediate conversion to open surgery for resection of the affected kidney (11); if intraoperative infection is not effectively controlled, it will progress to urosepsis, and severe cases may even be life-threatening (12). Thus, developing a standardized preoperative evaluation system to stratify stone complexity, improve surgical success, and reduce complications remains a key research focus.

To address the shortcomings of poor individual fit and fixed puncture points in traditional 3D-printed guides (15), we optimized the template design in three aspects. First, the D-shaped hollow structure was adopted to retain both the preset puncture channel and the operating space for the ultrasound probe, realizing dual positioning assistance of the template and ultrasound, which is the main reason for the shortened nephroscopic tract establishment time in the 3D-VR-Template group. Second, the puncture path was designed following the SVOF principle (16), selecting the posterior middle calyx near the Brodel's avascular plane as the target, which effectively reduces renal parenchymal injury and estimated intraoperative blood loss. Third, photosensitive resin was selected as the printing material, which maintains good dimensional stability after sterilization and has reliable biosafety (17–21), with advantages in production cost and cycle, providing feasibility for routine clinical application. The modified 3D-printed guide template shows potential application value in high-risk complex PCNL cases, but its clinical promotion still needs to be verified by larger sample studies.

Various navigation strategies have been widely applied in clinical PCNL practice, with distinct advantages and limitations. Conventional ultrasound guidance is the most widely used strategy with the advantages of low cost, no radiation and real-time operation, but it is highly operator-dependent and has limited spatial orientation for complex renal anatomy. Fluoroscopic guidance provides clear real-time visualization of guide wires and calculi, but it carries inevitable radiation exposure and has poor contrast of soft tissue. Electromagnetic navigation and augmented reality navigation can achieve high puncture accuracy with three-dimensional spatial positioning, but they require specialized expensive equipment and complex operation procedures, which greatly limit their popularization in primary hospitals.

The 3D-VR combined with 3D-printed guide template strategy in this study balances puncture accuracy, operation cost and clinical feasibility. It does not rely on large-scale navigation equipment, and can realize personalized preoperative planning and intuitive puncture guidance, which shows good application potential for high-risk complex stone cases, especially in medical centers without advanced navigation systems.

In this study, compared with low-to-moderate risk patients, high-risk patients in the conventional ultrasound-guided control group had statistically significant differences in the maximum cross-sectional area of calculi, operation time, and stone-free rate, which was consistent with the results of previous reports (22, 23), indicating that the S.T.O.N.E. score can predict the difficulty of surgery. High-risk patients had a larger average maximum cross-sectional area of calculi, greater calculus burden, greater difficulty with intraoperative lithotripsy, and longer lithotripsy time, resulting in longer operation time and a lower postoperative stone-free rate than low-to-moderate risk patients. Logistic regression showed that the total S.T.O.N.E. score and its sub-scores: S, T, and N were correlated with the calculus residue rate, while O and E were not correlated with the residue rate. This result was consistent with the report by Danis et al. (15), further indicating that larger calculi, longer nephroscope tract, and more involved calyces lead to greater surgical difficulty and more difficult calculus clearance, however, no statistically significant differences were found in estimated intraoperative blood loss, tract establishment time or hospital stay between different risk subgroups. This result is consistent with previous reports (24), suggesting that the S.T.O.N.E. score has limited predictive value for intraoperative indicators, which is related to its two-dimensional evaluation characteristics.

Several alternative explanations for the observed perioperative outcome differences should be considered. First, potential selection bias may exist due to the non-randomized study design: patients with more complex stones and higher clinical demand for precise puncture may be more inclined to choose 3D-assisted techniques. Although we have adjusted for major baseline confounders including stone size and hydronephrosis degree in multivariable models, residual confounding from unmeasured factors cannot be completely excluded. Second, all 3D-guided procedures were performed by a single senior surgeon who had mastered the application of 3D-printed guides, and the proficiency bias may also contribute to the improved outcomes; thus the results may not be directly generalized to novice surgeons. Third, differences in perioperative nursing and postoperative management, which were not included in the analysis, may also have an impact on the recovery indicators. Taken together, the efficacy observed in this study reflects an association rather than definitive causality, and high-quality randomized controlled trials are needed to further confirm the net effect of 3D-VR techniques.

In general, the above results suggest that the simple application of the S.T.O.N.E. score for preoperative evaluation of PCNL has good predictive value for stone-free rate and operation time, and among the sub-scores, S, T, and N have greater predictive value. However, the score has shortcomings in guiding surgical decisions, and the single application of the S.T.O.N.E. score cannot form a complete PCNL evaluation system. To address this problem, this study conducted additional research by introducing 3D virtual reconstruction technology to manufacture modified 3D guide plates during implementation. Given the additional cost of 3D-printed guide templates, the device was only offered to patients after full informed consent; all 19 patients who received the template were high-risk cases with S.T.O.N.E. score > 9. The results showed that, compared with high-risk patients in the conventional ultrasound-guided control group, the 3D-VR-Template group had better performance in estimated estimated intraoperative blood loss, nephroscope access establishment time, hospital stay, and stone-free rate. This was consistent with the report by Peng Hua et al. (25). This benefit is attributed to the predefined puncture path of the guide template, which shortens puncture time and avoids large intrarenal vessels. Therefore, the application of the 3D-printed guide plate in PCNL has significant advantages. At the same time, compared with high-risk patients in the simple 3D-VR group, there were no statistically significant differences in perioperative indicators between the 3D-VR-Template group and the simple 3D-VR group, except for a slight, non-significant difference in the stone-free rate. Given the small sample size of the template subgroup, larger studies are needed to explore potential differences between the two 3D-VR approaches. In addition, one of the major problems of 3D-printing is the high economic cost and long printing time. Long production times may delay surgery, while increased cost may be a barrier for some patients. Golab et al. (26) designed and manufactured a guide plate and successfully applied it during surgery. The puncture time was only 8 min, but the cost of 100 Euros and production time of 5 h and 35 min greatly increased the patient's economic burden and waiting time. Peng Hua et al. (25) designed and manufactured 10 puncture guide plates, with a production cost of 3,000 RMB per guide plate. Luo Houdong (27) et al. designed and manufactured models that required 1–2 days and a production cost of 4,000–5,000 RMB. Due to its unique material and shape design, the 3D-printing time for this study was 2 h, and the production cost was 1,900 RMB, offering advantages in terms of cost and time. This study found that the benefits of 3D-VR technology vary depending on the complexity of the patient's stones (as determined by STONE risk stratification). Logistic regression analysis further quantified this relationship: For high-risk, complex stones, 3D-VR-Template technology is an independent, favorable factor for improving stone-free rate, and its value far exceeds that of simple virtual programming or traditional ultrasound guidance. This strongly supports the need to integrate 3D-printed guide plates into the preoperative planning of complex PCNL.

This study also had certain limitations. This study also had several limitations. First, the overall sample size was moderate, and the 3D-VR-Template subgroup only included 19 cases, which may limit the statistical power of some secondary endpoints. Although *post-hoc* power analysis for the primary outcome (stone-free rate) in high-risk patients reached an acceptable level, the efficacy of the 3D-printed template in low-to-moderate risk patients could not be evaluated due to the absence of relevant cases. Large-scale multicenter studies are needed to further verify the conclusions. Second, potential indication bias (a type of selection bias) was present. The 3D-printed guide template was mainly applied to patients with high-risk complex stones based on clinical demand and patients' voluntary informed choice, leading to an uneven distribution of stone complexity across groups. To minimize the impact of this bias, we have adjusted for baseline confounders such as stone size and hydronephrosis degree in the multivariable regression model, but residual confounding cannot be completely excluded. Future well-designed randomized controlled trials with balanced baseline characteristics are warranted to confirm our findings. Third, all operations were performed by a single senior surgeon with extensive PCNL experience who had passed the learning curve of 3D-printed guide application. Thus, the results may not be directly generalized to novice surgeons, and the learning curve of this technique needs to be further evaluated. Fourth, only patients in the prone position were included, and the applicability of the 3D-printed template in other surgical positions (such as the supine position) and multi-tract PCNL remains to be verified. In addition, the impact of respiratory movement on puncture accuracy during surgery should also be considered in clinical application. Fifth, KUB radiography was used for postoperative stone evaluation in this study, which has slightly lower sensitivity for small radiolucent stones compared with non-contrast computed tomography. However, standardized imaging protocols and blinded independent evaluation were applied across all groups to minimize detection bias.

In conclusion, the application of 3D virtual reconstruction technology, combined with the S.T.O.N.E. score, can further reduce estimated intraoperative blood loss, shorten the patient's hospital stay, and improve the safety and success rate of surgery. At the same time, the modified 3D-printed guide plate is a new technology in PCNL. It further optimizes the therapeutic effect of renal calculi by improving intraoperative puncture accuracy and reducing estimated intraoperative blood loss, and by saving the patient's hospital expenses, which has great application value in PCNL.

## 5 Conclusion

1. The application of three-dimensional visualization virtual reconstruction technology combined with the S.T.O.N.E. score in PCNL can further reduce estimated intraoperative blood loss, shorten the hospital stay of patients, and is conducive to improving the safety and success rate of the surgery.
2. The modified 3D-printed guide plate-assisted percutaneous nephrolithotomy can optimize the PCNL pathway, enhance surgical accuracy, and reduce estimated intraoperative blood loss, which is worthy of clinical promotion and application.

## Data Availability

The original contributions presented in the study are included in the article/Supplementary Material, further inquiries can be directed to the corresponding author/s.
