# Supplementary material for: Three-dimensional virtual reconstruction combined with the S.T.O.N.E. score improves outcomes of PCNL: a multicenter study
Source: Front Surg. 2026 Sep 28;13:1880578. doi: 10.3389/fsurg.2026.1880578 (PMC13655103; doi:10.3389/fsurg.2026.1880578)
Supplement: Supplementary file 1 [file Datasheet1.docx]

Patient1

Patient2

Patient3

Patient4

Patient5

Patient6

Patient5

Patient7

Patient8

Patient9

Patient10

Patient11

Patient12

Patient13

Patient14

Patient15

Patient16
